# Rationale and design of the ADDITION-Leicester study, a systematic screening programme and Randomised Controlled Trial of multi-factorial cardiovascular risk intervention in people with Type 2 Diabetes Mellitus detected by screening

**DOI:** 10.1186/1745-6215-11-16

**Published:** 2010-02-19

**Authors:** DR Webb, K Khunti, B Srinivasan, LJ Gray, N Taub, S Campbell, J Barnett, J Henson, S Hiles, A Farooqi, SJ Griffin, NJ Wareham, MJ Davies

**Affiliations:** 1 Department of Cardiovascular Sciences, Victoria Building, Leicester Royal Infirmary, Leicester LE1 5WW, UK; 2 Department of Health Sciences, 22-28 Princess Road West, University of Leicester, LE1 6TP, Leicester, UK; 3 Department of Diabetes & Endocrinology, Victoria Building, Leicester Royal Infirmary, Leicester LE1 5WW, UK; 4 MRC Epidemiology Unit, Institute of Metabolic Science, Box 285, Addenbrooke's Hospital, Hills Road, Cambridge, CB2 0QQ, UK

## Abstract

**Background:**

Earlier diagnosis followed by multi-factorial cardiovascular risk intervention may improve outcomes in Type 2 Diabetes Mellitus (T2DM). Latent phase identification through screening requires structured, appropriately targeted population-based approaches. Providers responsible for implementing screening policy await evidence of clinical and cost effectiveness from randomised intervention trials in screen-detected T2DM cases. UK South Asians are at particularly high risk of abnormal glucose tolerance and T2DM. To be effective national screening programmes must achieve good coverage across the population by identifying barriers to the detection of disease and adapting to the delivery of earlier care. Here we describe the rationale and methods of a systematic community screening programme and randomised controlled trial of cardiovascular risk management within a UK multiethnic setting (ADDITION-Leicester).

**Design:**

A single-blind cluster randomised, parallel group trial among people with screen-detected T2DM comparing a protocol driven intensive multi-factorial treatment with conventional care.

**Methods:**

ADDITION-Leicester consists of community-based screening and intervention phases within 20 general practices coordinated from a single academic research centre. Screening adopts a universal diagnostic approach via repeated 75g-Oral Glucose Tolerance Tests within an eligible non-diabetic population of 66,320 individuals aged 40-75 years (25-75 years South Asian). Volunteers also provide detailed medical and family histories; complete health questionnaires, undergo anthropometric measures, lipid profiling and a proteinuria assessment. Primary outcome is reduction in modelled Coronary Heart Disease (UKPDS CHD) risk at five years. Seven thousand (30% of South Asian ethnic origin) volunteers over three years will be recruited to identify a screen-detected T2DM cohort (n = 285) powered to detected a 6% relative difference (80% power, alpha 0.05) between treatment groups at one year. Randomisation will occur at practice-level with newly diagnosed T2DM cases receiving either conventional (according to current national guidelines) or intensive (algorithmic target-driven multi-factorial cardiovascular risk intervention) treatments.

**Discussion:**

ADDITION-Leicester is the largest multiethnic (targeting >30% South Asian recruitment) community T2DM and vascular risk screening programme in the UK. By assessing feasibility and efficacy of T2DM screening, it will inform national disease prevention policy and contribute significantly to our understanding of the health care needs of UK South Asians.

**Trial registration:**

Clinicaltrial.gov (NCT00318032).

## Introduction

Type 2 Diabetes Mellitus (T2DM) is an increasingly common, potentially devastating disease characterised by prolonged asymptomatic hyperglycaemia and insidious vascular complications [1]. At present, 50% of new T2DM cases have demonstrable atherosclerosis at diagnosis and glucose abnormalities commonly characterise acute coronary and cerebro-vascular thrombotic events in people not known to have the disease [2,3]. Recent outcome studies suggest intensive glucose control among people with long-standing T2DM is associated with limited reduction in cardiovascular events, whereas sustained optimisation earlier in the trajectory of the disease may be associated with significant micro and macro-vascular benefits [4-6].

### Screening for T2DM

The frequency of T2DM, its latent presentation and potentially preventable burden of complications make it an attractive target for earlier identification and intervention through screening [7,8]. Despite convincing rationale there is in fact little evidence that this approach improves T2DM outcomes, or that treatment effective for conventionally diagnosed cases produces greater benefit if commenced within the lead time between detection by screening and clinical diagnosis [9]. Furthermore the continuous relationship between glucose concentration and cardiovascular disease well below the diagnostic threshold for T2DM [10] suggests screening programmes should include non-diabetes range hyperglycaemia (Impaired Fasting Glycaemia and Impaired Glucose Tolerance) if they are to improve population level outcomes. Primary prevention studies in these groups result in weight loss and slow progression to diabetes [11,12] supporting the concept of earlier identification and lifestyle intervention for those at risk of T2DM.

It is currently unclear how effective population screening will be at identifying people with T2DM or whether incorporating a mix of cardio-metabolic factors, including non-diabetes range hyperglycaemia, into screening programmes will substantially increase the yield of individuals at high cardiovascular risk [9]. There are additional concerns with respect to the potential adverse consequences of screening [13,14], its cost-effectiveness [15] and the magnitude of achievable cardiovascular risk reduction within this largely symptom-free population [16].

### Black and Minority Ethnic (BME) groups

Certain ethnic groups in the UK are at particularly high risk of abnormal glucose tolerance and T2DM, with reported prevalence 2-6 times that of the background white European population [17,18]. Speculated higher than average T2DM progression rates amongst UK south Asians are supported by significant global variation in reported incident cases but robust prospective data is lacking within Westernised ethnic minority populations. Studies gauging reaction to T2DM screening within these groups is also limited [19] but suggests response rates are more likely to be influenced by cultural beliefs, social stigma attached to certain conditions, and the attitude of the local community to western health care methods [20,21]. It is essential barriers to screening activity together with the effort required to overcome them are quantified if they are to inform effective planning and implementation of culturally sensitive interventions [22].

### The ADDITION study

As a result of such critical uncertainties the UK National Screening Committee currently recommend a targeted rather than universal approach, with screening confined to groups at particularly high risk of T2DM [23,24]. There is some evidence that UK general physicians are increasingly carrying out opportunistic or planned community screening of their patients [25,26]. Implementation of the National Health Service (NHS) vascular check programme recommending glucose testing for 40-70 year olds will undoubtedly further increase T2DM screening activity in the UK [27].

Priority should be directed towards developing a robust evidence base informing national policy and protecting against indiscriminate, poorly coordinated screening programmes. The results of randomised controlled trials among screen-detected cases with outcomes assessing vascular complications, health satisfaction, process of care indicators and cost are vital to this process.

ADDITION (Anglo-Danish-Dutch Study of Intensive Treatment in People with Screen Detected Diabetes in Primary Care) is a multi-centred randomised controlled trial evaluating the effectiveness of multi-factorial treatment on risk of cardiovascular disease events among over 2500 patients with screen-detected diabetes [28-30]. ADDITION-Leicester contributes to this multi-centre study but is also a stand-alone trial evaluating screening within a UK multiethnic group and quantifying the efficacy of optimised treatments on modelled cardiovascular risk over five years.

This paper describes the aims and methods of both screening and intervention phases of the ADDITION-Leicester study.

## ADDITION-Leicester Objectives

The primary aim of the ADDITION-Leicester study is to evaluate the efficacy and cost-effectiveness of 1) a universal screening programme for T2DM and 2) intensive multi-factorial cardio-protection in those identified with T2DM within a UK multiethnic population. This objective will be achieved by determining the feasibility of screening as defined by the uptake and yield achievable within a primary care setting with a known 20-30% ethnic minority (Indo-Asian) presence. The health service and patient costs of screening for T2DM and other glucose abnormalities will be evaluated, together with objective assessments of five-year cardiovascular risk and mortality. ADDITION-Leicester is registered with ClinicalTrials.gov with identifier NCT00318032.

The study consists of two phases a screening phase, employing a universal gold standard diagnostic test for T2DM and an intervention phase, delivering a randomised trial of structured cardiovascular risk intervention in screen-detected cases.

A third element, the ADDITION-Leicester pre-diabetes cohort study prospectively assesses non-diabetes range fasting and post-challenge hyperglycaemia. The aim of the ADDITION-Leicester Prediabetes Cohort Study is to determine the annual rate of progression over five years of follow up and to characterise T2DM susceptibility phenotypes.

### Methods/Design

ADDITION-Leicester adopts a community based non-selective screening approach within a representative cluster of General Practices. The study is coordinated from a regional academic centre hosted by the University of Leicester and University Hospitals of Leicester, NHS Trust but delivered in primary care through an established diabetes research network (South East Midlands Diabetes Research Network). The study is supported by competitive Department of Health project and NHS Support for Science grants. Ethical approval was obtained from the University Hospitals of Leicester (UHL09320) and Leicestershire Primary Care Research Alliance (64/2004) local research ethics committees. The study was conducted in accordance with the principles of the 1996 Helsinki Declaration. Written informed consent was obtained for all participants involved in both phases of ADDITION-Leicester study at the time of diabetes screening.

#### Study location

Volunteers were recruited from general practices in urban, suburban and rural Leicestershire, England, United Kingdom. Screening focuses upon Leicester, the county capital with an estimated population of 279,921, an ethnic minority prevalence of 32% (82% Gujarati speaking first or second generation Indo-Asians) and a local authority ranked amongst the twenty most deprived in the United Kingdom (2006 census data available at http://www.leicester.gov.uk).

### Phase 1: The screening phase

#### Identification of an eligible population: practice data handling and electronic mailers

Clinical leads from the 46 General Practices forming the Leicestershire and Rutland Strategic Health Authority were approached to participate in ADDITION-Leicester. Personalised letters were sent to the practice manager, partners and nursing staff of each practice reiterating the importance of the study to primary care, the roles of individual practices, the required commitment and the availability of remuneration for all incurred costs. A principal investigator and member of the research team visited interested practices to discuss the study in detail. 28 practices responded positively with consent for an initial database search using an extraction programme compatible with the widely used clinical EMIS (Egton Medical Information Systems Ltd, York UK) system. This specialised software generates an anonymised Master Practice List (MPL) that matches individual data to a random unique identifier (a six-digit and single letter ADDITION-Leicester number). An MPL representative of the practice population is considered essential for further participation in the study and eight practices were excluded at this stage due to data extraction failure or search software incompatibility. Practices were considered eligible if the MPL captures more than 70% of the total practice population. The MPL captures practice population demographics (age, sex, occupation, medical history, active prescriptions) and known T2DM frequency, enabling future comparison of responder/non-responder characteristics and total T2DM disease prevalence. Applying the study criteria to the MPL produces an eligible population list which is reunited with the practice dataset to provide personal details necessary to post an invitation for screening (first mailer). Those meeting the inclusion criteria are sent details of the study along with a returnable request for culturally appropriate information written in five major South Asian languages (Hindi, Gujarati, Bengali, Urdu and Punjabi). Having expressed an interest in the study, potential participants are sent individual screening appointments at either a hospital site or a mobile screening unit located within their community. Non-responders are sent a second invitation (second mailer) within six months. To ensure confidentiality is maintained practice staff handle initial database searches and mailing tasks. South Asian ethnicity is defined at this stage by forename and surname mapping using specialised software developed from census data (Nam Pehchan) [31]. Mean practice deprivation scores are calculated from individual MPL data using an Index of Medical Deprivation (IMD 2007) [32]

**Figure 1:**
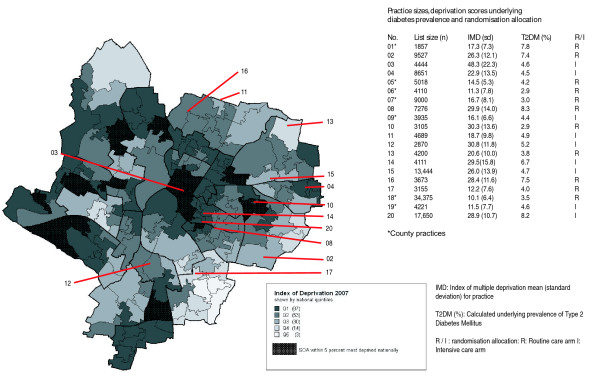
**Leicester City wards by quintiles deprivation (IMD2007)**.

The size, geographical location and deprivation status of the 20 practices participating in ADDITION-Leicester is shown in Figure 1. Our calculated mean practice IMD scores match national survey deprivation quintiles and depicted practices within the Leicester city boundary appear typical of an urban UK deprivation distribution. To ensure all 20 sites are covered within the study timeframe, in six practices the entire eligible population have been sent information regarding the study whilst a random sample of the population are included in the remaining practices.

Inclusion and exclusion criteria for the study are similar to the multi-centre ADDITION-Europe study [28]. Inclusion criteria are white Europeans between the ages of 40-75 years and south Asians, Afro-Caribbean's and other races between the ages of 25-75 years. A lower age cut-off for BME groups was chosen due to the reported higher risk of T2DM. People with the following pre-existing conditions are excluded (general practice diagnosis and database recorded), T2DM, terminal illnesses with a likely prognosis of less than 12 months, psychiatric illness likely to hinder informed consent, pregnancy and lactation.

#### Screening visit measurements

Standardised procedures are in place across screening sites to ensure universal gold-standard diagnostic testing for T2DM, IFG and IGT [33]. Individuals are asked to fast for eight hours prior to attending a screening appointment and to bring a list of prescribed medications with them. Before beginning the over night fast participants are asked to consume their regular evening meal and snacks, but refrain from alcohol consumption. At baseline (V0) and annual pre diabetes cohort screening visits a standard 75g oral glucose tolerance test (OGTT) is undertaken following informed consent. This test is postponed if in the preceding three days instructions to follow a normal unrestricted diet are not followed or participants report fever or unusual physical activity. On the day of testing prescribed morning medications are permitted but participants are asked not to run to their appointment or smoke until after the test. Plasma samples are obtained immediately before (fasting plasma glucose FPG) and 120 minutes after the glucose challenge (two hours post challenge glucose 120-PG) along with fasting samples for serum urea and electrolytes (UE), liver function tests (LFT), lipids (total cholesterol, LDL-cholesterol, HDL-cholesterol, triglycerides), HbA1c% (glycosylated haemoglobin) and renal function (creatinine and modification of diet in renal disease estimated glomerular filtration rate (MDRD eGFR)) (table 1). A spot urine sample for urinalysis and albumin excretion rate is also collected.

**Table 1:** Summary of assessments performed at Baseline (V0), annual pre diabetes and randomised controlled trial (RCT) visits (V1-5) of the ADDITION-Leicester study

Visit	Baseline Screening (v0)	Pre diabetes annual cohort	T2DM RCT *Year 1 (v1)* *intensive/* *routine*	T2DM RCT *Year 2 (v2)* Intensive	T2DM RCT *Year 3 (v3)* Intensive* /routine	T2DM RCT *Year 4(v4)* intensive only	T2DM RCT *Year 5 (v5)* *intensive/* *routine*
***Medical Procedures:***							
Blood Pressure	**√**	**√**	**√**	**√**	**√**	**√**	**√**
Electrocardiogram (ECG)	**√**	**√**	**√**	**√**	√*	**√**	**√**
Foot Check	-	-	**√**	**√**	√*	**√**	**√**

***Biochemical measurements:***							
75g-OGTT: Fasting & 120 min glucose	**√**	**√**	**-**	**-**	**-**	**-**	**-**
UE, LFT, Lipid profile, HbA1c%	**√**	**√**	**√**	**√**	**√**	**√**	**√**
Renal function & urine ACR	**√**	**√**	**√**	**√**	**√**	**√**	**√**
TFT	**-**	**-**	**√**	**√**	√*	**√**	**√**

***Anthropometric measurements:***							
Height	**√**	**√**	**√**	**√**	**√**	**√**	**√**
Weight	**√**	**√**	**√**	**√**	**√**	**√**	**√**
Hip/Waist circumference	**√**	**√**	**√**	**√**	√*	**√**	**√**
Bioimpedence (% body fat)	**√**	**√**	**√**	**√**	√*	**√**	**√**
Body Mass Index (BMI)	**√**	**√**	**√**	**√**	**√**	**√**	**√**

Screening Questionnaires*:							
Medical/family history/medications	**√**	**√**	**√**	**√**	√*	**√**	**√**
Alcohol/smoking status	**√**	**√**	**√**	**√**	√*	**√**	**√**

***Self-reported Questionnaires:***							
Findrisc[45], Cambridge risk scores[46]	**√**	**√**	**√**	**√**	√*	**√**	**√**
EuroQol, EQ-5D[47]	**√**	**√**	**√**	**√**	√*	**√**	**√**
WHO-5, BFI 44[48]	**√**	**√**	**√**	**√**	√*	**√**	**√**
IPAQ[49], Berlin ESS[50]	**√**	**√**	**√**	**√**	√*	**√**	**√**
Michigan neuropathy[51]	**√**	**√**	**√**	**√**	√*	**√**	**√**
T2DM: Life Quality/treatment satisfaction	**-**	**-**	**√**	**√**	**√**	**√**	**√**
ADDQoL[52], DTSQ[52], W-BQ 28[52]							

***Arterial measurements sub study:***							
_cf_PWV, PCA[36,37]	**√**	**√**	**√**	**-**	**-**	**-**	**√**

***Biobank storage aliquots:***							
8 × 2 ml Plasma(4), serum (4),	**√**	**√**	**√**	**√**	√*	**√**	**√**

***Genetic Sample:***							
Whole blood (EDTA)	**√**	**-**	**-**	**-**	**-**	**-**	**-**

Key:

OGTT: 75 g Oral Glucose Tolerance Test (preparation as per WHO expert consensus report -1999)

UE: Biochemistry Urea & Electrolytes panel ACR: Albumin:Creatinine Ratio

LFT: Liver Function Tests (Bilirubin, alanine transaminase, alkaline phosphatase, gamma glutamyl transpeptidase)

HbA1c%: Glycosylated Haemoglobin

Lipid profile: Total, LDL, HDL Cholesterol & Triglycerides

TFT: Thyroid Function Test (Thyroid Stimulating Hormone, free thyroxine T4)

_cf_PWV, PCA: carotid-femoral Pulse wave Velocity Pulse Contour Analysis (Photoplethysmography derived)

Screening Questionnaires*: Completed during Interview with trained research nurse

Anthropometric measurements are performed by trained staff following standard operating procedures, with height being measured to the nearest 0.1 cm using a rigid stadiometer and weight in light indoor clothing measured to the nearest 0.1 kg with a Tanita scale (Tanita, Europe). Body fat percentage is measured via calibrated bio impedance (Tanita, Europe). Body mass index (kgm^-2^) is defined as weight in kilograms divided by height in metres squared. Waist circumference was measured at the mid-point between the lower costal margin and the level of the anterior superior iliac crest to the nearest 0.1 cm.

Brachial blood pressure is measured three times using standardised Omron M7 digital sphygmomanometers (Omron Healthcare, Milton Keynes, UK) with the participant in a seated position. An average of the second and third readings is recorded as per British Hypertension Society guidelines [34] with written instructions for abnormal readings. A 12 Lead electrocardiogram (ECG) is performed using a Nihon Kohden CardioFax Gem machine (Nihon Kohden Europe GmbH, Rosbach vor der Höhe, Germany). An in-house physician interprets ECGs on the day of the visit, codes for ischaemia and left ventricular hypertrophy [35] and reports back to the general practitioner.

Self-completed questionnaires are used to assess baseline smoking status, alcohol consumption, occupation, and ethnicity. Validated questionnaires measuring physical activity, sleep quality, risk of diabetes, neuropathy, and diabetes-specific psychological domains of well being and anxiety are also included (table 1). All measurements are performed by a dedicated team of research nurses trained to document relevant medical information and family history on a standardised case report form during a 20 minute one to one interview on the day of screening. The clinical team are unaware of participants study group allocation or glucose diagnosis.

At major visits (V0-V4, and pre diabetes assessments) further venepuncture is performed for future biomarker research (table 1). Consent is obtained for the -80°C storage of multiple anonymised serum, plasma and whole blood aliquots. These samples contribute to a biobank facilitating translational research exploring the pathogenesis of insulin resistance, vascular complications and genetics of T2DM.

An option for further physiological measurements is incorporated as a sub study amendment at visits V0, V1 and V4. Volunteers consent to return on a separate occasion for non-invasive arterial assessments and blood tests. Trans-cutaneous ultrasonic Pulse Wave Velocity (_cf_PWV), and digital photoplethysmographic pulse contour analysis (PCA) are performed under controlled conditions using commercially available equipment (PT4000, Cardinal Healthcare, Basingstoke, UK) [36,37].

#### Diagnosis and reporting

Results are relayed via written correspondence and copied to participant and general practitioner. All biochemical measurements are performed in house at the University Hospitals of Leicester NHS trust. Glucose samples are taken in fluoride oxalate test tubes and placed immediately in a portable 4 litre 4°C refrigerator (also available on board the mobile screening unit). HbA1c% is analysed by a DCCT aligned Biorad Variant HPLC II system (Bio-Rad laboratories, Hemel Hempstead, UK). The imprecision coefficient of variation of this machinery is <0.1%, the reference intervals fit with national recommendations valid for carriers of variant Hb S, C and Q. Samples are processed within a maximum of two hours, using an Abbott Aeroset clinical chemistry analyser (Abbott laboratories, Maidenhead, UK), which employs the hexokinase enzymatic method. This machinery has an imprecision coefficient of variation of 1.61%. Serum total cholesterol, HDL-cholesterol, LDL-cholesterol and triglycerides are measured by means of enzymatic techniques (Dade Behring Dimension analyser, Newark, USA). Plasma creatinine is analysed with kinetic colorimetric methods. Plasma levels of urea and electrolytes, bilirubin, alanine aminotransferase, alkaline phosphatase and thyroid stimulating hormone are analysed by means of the Dade Behring Dimension analyser.

**Figure 2:**
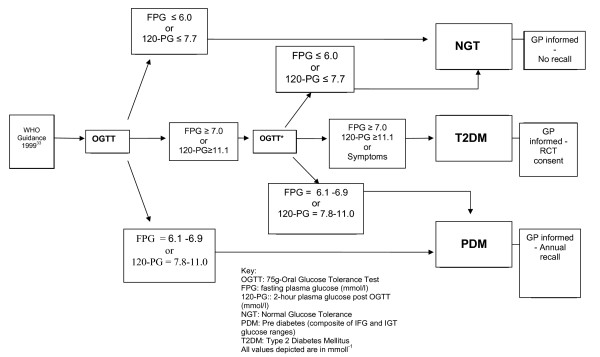
**ADDITION-Leicester algorithm for the diagnosis of pre-diabetes and screen-detected T2DM**.

Participants are categorised according to World Health Organisation (WHO) criteria [33]. Diabetes is defined as a fasting blood glucose of greater or equal to 7 mmoll^-1 ^and/or 120-PG of greater than or equal to 11.1 mmoll^-1^. Impaired Fasting Glycaemia (IFG) is defined as a fasting blood glucose concentration of between 6.1 and 6.9 mmoll^-1 ^inclusive and IGT as a blood glucose concentration of between 7.8 and 11 mmoll^-1 ^inclusive. Impaired Glucose Regulation (IGR) is defined as any combination of IFG and/or IGT. The diagnosis of T2DM is confirmed by an in house physician on the basis of two abnormal glucose results obtained on separate visits, unless hyperosmolar symptoms suggestive of hyperglycaemia are reported at the screening visit. Asymptomatic individuals with a diabetes range OGTT are asked to maintain their current lifestyle and return for a confirmatory test (re screen) within one week. For a diagnosis of T2DM in asymptomatic individuals, one positive result from either a fasting and/or 120-PG is required on both visits. In the event of discordant OGTT results (eg. baseline diabetes followed by rescreen IGR) participants are categorised as having IGR (figure 2). Volunteers diagnosed with diabetes are entered into a cluster randomised controlled trial of multi-factorial cardiovascular risk intervention whilst those identified with IGR are given lifestyle advice and invited to join the ADDITION-Leicester prediabetes cohort study.

### ADDITION-Leicester Pre diabetes cohort study

Non-diabetes range asymptomatic hyperglycaemia is of clinical relevance to screening programmes due to the associated increased risk of T2DM and cardiovascular disease. Volunteers found to be within fasting or post-challenge glucose categories of IFG and IGT (collective lay-term prediabetes) at baseline are provided with written lifestyle advice and invited for annual screening. The ADDITION-Leicester prediabetes cohort study annual screening protocol is identical to the baseline visit (table 1), all results are relayed to participant and general practitioner and those with diabetes range results are recalled for a second glucose tolerance test. The process for continued follow up differs however, as newly diagnosed T2DM is considered an endpoint and the case is returned to the care of the primary care specialist rather than entering the trial phase. Continued Prediabetes or normal glucose tolerance range results are invited for further annual assessments.

### Phase 2: A randomised controlled trial of multifactorial intervention in individuals with screen-detected T2DM

Phase 2 is a pragmatic, cluster randomised, parallel group trial among people with screen-detected T2DM comparing intensive multi-factorial treatment with routine care in general practice according to national guidelines.

Randomisation is performed by an independent committee provided with practice demographics, deprivation status and approximate T2DM prevalence of individual practices. Practices and not individuals are randomised via a minimisation procedure with a 1:1 ratio. The screen detected T2DM control group (routine care arm) receive "usual care" within the primary care setting, according to national recommendations for management of T2DM and cardiovascular disease [38]. These participants will be reviewed one and five years post diagnosis, when anthropometric and biochemical data will be collected. The screen detected T2DM intervention group (intensive care arm) are introduced to dedicated, specialist physicians and nurses who provide a structured, intensified, protocol-driven, multi-factorial approach again within the primary care setting (table 2).

**Table 2:** ADDITION-Leicester algorithm for the management of hyperglycaemia, hypertension and dyslipidaemia.

	Basic Treatment TARGET	add if above TARGET	add if above TARGET +BMI>19 or creatinine>130	Supplementary treatment If still above TARGET	
Blood Glucose	***HbA1c <6.5%*** SMBG Tuition DESMOND Dietary Advice	***HbA1c >6.5%*** SMBG Tuition DESMOND Biguanide	***HbA1c >6.5%*** SMBG Tuition DESMOND Insulin	***HbA1c >6.5%*** Biguanide Sulphonylureas Thiazolidinediones THEN Stop TZD Add basal Insulin (bedtime)	***HbA1c >6.5%*** ***and on *** ***Biguanide*** ***Sulphonylureas*** ***Insulin*** (basal/bolus) Intensify & titrate Insulin

Blood Pressure	***BP *** ***< 130/80 mmHg*** No treatment	***BP *** ***>130/80 mmHg*** ACE	***BP >135/80 mmHg*** ARB CCB Thiazide	***BP *** ***>130/80 mmHg*** ACE ARB/CCB	Thiazide alpha/beta Blocker

Cholesterol	***<3.5 mmol/L*** Diet	**>*3.5 mmol/L*** ***TG >6.0 mmol/L*** Diet Statin Consider Ezetimibe/Fibrate			

Aspirin	75 mg to all patients, unless contraindications of gastrointestinal bleeding, ulcers or haemophilia. If Aspirin contraindicated, consider Clopidogrel.				

KEY

**ACE**: Perindopril 2-4 mg daily or Ramipril 2.5-10 mg daily

**SMBG**: self-monitored blood glucose

**Biguanide: **Metformin 1-2 g daily

**Sulphonylurea: **Gliclazide MR 30-120 mg daily or Glimepiride 1-4 mg daily

**Insulin: **Basal analogue: Glargine. Short-acting analogue: Novorapid or Premixed: Novomix30 twice daily

**Thiazolidinedione: **Pioglitazone 30-45 mg daily

**ARB: **Angiotensin receptor blocker: Losartan 25-50 mg daily

**CCB: **Calcium Channel Blocker: Amlodipine 5 mg daily

**Thiazide: **Bendrofluazide 2.5 mg daily

**Alpha/beta blocke**r: Doxazosin MR 4-8 mg or Bisoprolol 5-10 mg daily

**Statin: **Simvastatin 20-40 mg or Atorvastatin 20-40 mg daily

**Fibrate: **Fenofibrate (micro) 267 mg or Bezafibrate MR 400 mg daily

#### Routine care arm intervention

Screen-detected T2DM cases entering the control group (routine care arm) receive the standard of care normally provided by their primary health care team. Within 24 hours of diagnosis a letter detailing the results of screening is faxed to the practice, the participant is informed (where possible via telephone contact) and an urgent appointment with their general practitioner advised.

At the time of randomisation each practice is sent a copy of and electronic links to Leicestershire evidence based guidelines adapted from national recommendations for diabetes care http://www.leicestershirediabetes.org.uk/[38]. At the time of writing these included local targets and protocol considered appropriate for effective cardiovascular risk management in T2DM. Specific local recommendations advise a glycosylated haemoglobin (HbA1c%) of <7.0%, blood pressure of <140/85 mmHg and a serum total cholesterol of <4.0 mmoll^-1^. Systematic evidence-based standards of care are expected of all UK general practices engaged in delivering diabetes services as outlined in the NHS National Service Framework for Diabetes [39]. All participating practices are also performance managed by active participation in the quality outcomes framework for diabetes [40].

The routine care arm will be reviewed one and five years post diagnosis when only anthropometric and biochemical data will be collected. The intensive treatment team do not engage in the management of these patients and general practitioners are asked to follow usual referral procedure if specialist advice or intervention is required.

#### Intensive care arm intervention

Care for the intensive arm is based upon a paradigm of multi-factorial intervention shown to improve mortality in T2DM [41]. Structured education (diabetes education and self-management for ongoing and newly diagnosed diabetes - DESMOND [42]) is initially offered to all patients in the intensive arm with attendance ideally, within the first two months of diagnosis. Sessions are delivered by two trained educators and aim to facilitate lifestyle changes in relation to dietary habits, physical activity levels, smoking cessation and glucose monitoring. Those participants who are unable, or decline the opportunity to attend the structured education programme are offered one-to-one advice with a dietitian. All volunteers are offered a glucometer, and encouraged to maintain a reflective diary. On-going professional support is provided in the first year through an individualised peripatetic clinic offering two monthly visits from a diabetes specialist nurse or physician. Ultimately, participants are encouraged to self-manage their diabetes by identifying personalised goals which facilitate individualised behaviour and lifestyle change.

Patients without specific contra-indications are advised to take aspirin 75 mg orally and prescribed lipid lowering therapy (simvastatin 40 mg once daily) if total cholesterol concentration exceeds 3.5 mmoll^-1^. An individualised, stepwise approach to management according to specified algorithms is adopted to ensure optimisation of hypertension, dyslipidaemia and hyperglycaemia according to protocol-driven targets using medication within existing licensed indications (table 2). Recommended drug choices take in to account treatment efficacy, side-effects and cost, the main priority being achievement of treatment targets whilst maintaining flexibility and low rates of adverse events. The approach is deliberately pragmatic with the final decision on choice of medication determined by the health care professional and patient. Treatment targets for the intensive care arm are based on complex interventions with proven efficacy in T2DM; HbA1c <7.0% with initiation of treatment at 6.5%, blood pressure <130/80 mmHg, and total cholesterol <3.5 mmoll^-1 ^[41]. After the first year, community visits are extended to every four months but continue to be guided by protocol driven blood pressure, HbA1c%, and lipid targets.

At annual visits (V1-V4 table 1) interim outcome measures and additional biomedical assessments are performed, including urinary albumin creatinine ratio (ACR), electrocardiography and thyroid function tests. This visit includes a standardised foot examination incorporating a vascular doppler assessment, ankle-brachial pressure indices and monofilament neuropathy testing. A stereoscopic digitalised retinal examination is performed and independently verified by operators blinded to the participants study group allocation.

The intensive treatment protocol is externally moderated by the trial steering committee and specifically designed to achieve HbA1c%, blood pressure and serum lipid targets below current national recommendations.

### Endpoints and outcomes

#### Primary endpoint

The primary endpoint is reduction in modelled coronary heart disease at five years using the United Kingdom Prospective Diabetes Study (UKPDS CHD) risk equation [43]. The UKPDS CHD risk engine has the advantage of being diabetes specific and incorporates an adjustment for the effects of south Asian ethnicity when calculating CHD risk. It has recently been shown to be as accurate as other CHD risk assessment tools in T2DM and has been validated for the effects of ethnicity [43].

#### Secondary and intermediate outcomes

Secondary outcomes of ADDITION-Leicester are a microvascular complication composite of diabetic retinopathy, neuropathy or microalbuminuria, a vascular atherosclerosis surrogate (carotid femoral pulse wave velocity), all cause mortality (assessed by participants tagging with the Office for National Statistics), non-fatal cardiovascular events, hospitalisation, health service cost and quality of life indicators.

Intermediate outcomes are measured annually in the intensive care arm and at one, three and five years in the routine care arm (table 1). These include HbA1c%, blood pressure, total cholesterol, microalbuminuria, self-reported hypoglycaemic episodes, weight, physical activity, ankle-brachial pressure indices and smoking status.

#### Screening outcomes

Response and attendance characteristics (obtained from MPLs) will provide an objective assessment of screening within the invited population. The programme is of sufficient size to enable the feasibility and complexities of screening to be stratified by ethnicity and socio-economic status. Outcomes will include the number of individuals responding to first and second mailers, presenting for screening, and subsequently diagnosed with T2DM or IGR. Metabolic dysfunction, cardiovascular risk, psychological status and self-perceived health in newly diagnosed T2DM and IGR will be determined. Population effects of screening will be determined via Office of National Statistics (ONS) mortality tagging and self-report health questionnaires five years after screening. Response and attendance rates will also be compared with previous population T2DM screening studies to enable specific conclusions to be drawn about the ADDITION-Leicester population [25,29].

#### Health economic outcomes

The economic analysis will establish the NHS costs of Phase 1 (screening) for T2DM and IGR from a patient and health service perspective. The cost-effectiveness of multifactorial intervention within our population of screen-detected cases will be determined from a health service perspective. Personal patient costs to attend the assessments (screening and as part of the trial) are collected at each visit. Health service use is assessed using data on consultations with healthcare professionals, hospitalisations and medications in the twelve months leading up to annual review.

### Statistical methods and power calculation

Assuming a prevalence of screen-detected diabetes of 4.5%, we calculated a target of 7,000 (30% (2,100) South Asians) volunteers over three years sufficient to identify a screen-detected T2DM cohort (n = 225) demonstrating a 6% difference (80% power, alpha 0.05) between routine and intensive groups at one year assuming an intra cluster correlation coefficient of 0.14 and allowing for a loss to follow-up of 15%.

The benefits of screening and intensive treatment will be assessed using an intention to treat and allowing for clustering of patients by practice. The UKPDS CHD score at 5 years will be compared along with all secondary outcomes by treatment group, adjusting for differences in baseline variables. Estimates will be presented with 95% confidence intervals to reflect uncertainty in estimations. Sensitivity analysis assuming a range of outcomes for non-completers will be informed by baseline data. The primary perspective for the cost analysis will be the health service, with personal costs as a secondary perspective. The costs of intensive intervention will be compared with unit change in health utility at one year. Costs at one year and future costs derived from existing data will be compared with modelled risk of death and cardiovascular events, with appropriate sensitivity analysis. The cost of screening and the screening plus intensive treatment will be compared with changes in health utility from questionnaire data.

#### Data entry

Source data, CRF and Questionnaires, are entered by Abacus Data and Document Capture LTD (Luton, UK) using double data entry to ensure acceptable accuracy and validation. Data discrepancies are handled by a small team of experienced researchers with clinical input where necessary (levels of agreement >90% discrepancies settled by third adjudicating physician).

### Funding and timescale

The project is funded for support and treatment costs by NHS Department of Health Support for Science and project grants. The screening phase of ADDITION-Leicester is now complete, having invited over 35,000 volunteers and identified 345 new cases of T2DM. As the intervention is delivered in tandem with the screening phase, the last trial visit is forecast for May 2012 assuming a mean follow up of 5 years.

## Conclusion

Earlier identification of hyperglycaemia through screening may be an effective way of improving vascular outcomes in people with T2DM. Although modelling studies suggest screening is cost-effective [44] the implications of implementing major screening programmes are of such magnitude that a sound evidence base is essential before expert consensus can be reached. We have described the methodology and design of a large scale population based screening programme and randomised controlled trial of newly diagnosed T2DM cases. To our knowledge ADDITION-Leicester is the largest screening study focusing upon a major ethnic minority at increased risk of T2DM. The study aims to comprehensively describe glucose status (via a glucose tolerance test) and cardiovascular risk at a population level as well as describing the practicalities, cost effectiveness and overall feasibility of cardiovascular risk screening within this group.

The results will be of major relevance to screening policy makers and those charged with delivering frameworks for effective chronic disease management in primary care. Of particular importance is the emphasis on south Asians a major yet under researched high risk ethnic minority group.

## List of Abbreviations

ADDITION: (Anglo-Danish-Dutch Study of Intensive Treatment in People with Screen Detected Diabetes in Primary Care); DESMOND: Diabetes Education and Self-Management for Ongoing and Newly Diagnosed diabetes; T2DM: Type 2 Diabetes Mellitus; UK: United Kingdom; RCT: Randomised Controlled Trial; MPL: Master Patient List; OGTT: Oral Glucose Tolerance Test; IFG: Impaired Fasting Glycaemia; IGT: Impaired Glucose Tolerance; HbA1c%: Glycosylated Haemoglobin; GP: General Practitioner; ONS: Office of National Statistics, UK; UE: Urea & Electrolytes; LFT: Liver Function Tests; TFT: Thyroid Function Tests; BMI: Body Mass Index (Kgm^-2^); eGFR: estimated Glomerular Filtration Rate; ACR: Albumin Creatinine Ratio; UKPDS: United Kingdom Prospective Diabetes Study; ACE: Angiotensin Converting Enzyme Inhibitor

## Competing interests

MJD has received funds for research, honoraria for speaking at meetings and has served on Advisory Boards for Lily, Sanofi Aventis, MSD and Novo Nordisk. KK has received funds for research, honoraria for speaking at meetings and served on Advisory Boards for Astra Zeneca, GSK, Eli Lily, Novartis, Pfizer, Servier, Sanofi Aventis, MSD and Novo Nordisk. SG has received speaker fees from Eli Lilly, GSK, MSD, Colgate Palmolive and Unilever; and research support from Novo Nordisk. NJW has served on advisory panels for Unilever and GlaxoSmithKline; and received support from Novo Nordisk All other authors have nothing to declare

## Authors' contributions

DW, BS, SC, JH and JB comprised the clinical team running the RCT and screening phases. DW produced the manuscript and was clinical lead, whilst KK, MJD, SG and NW conceived the study. MJD (Principal Investigator) and KK designed the study protocol. NT and LG performed statistical analyses, database management and contributed to the manuscript. SH was an interim project manager whilst SG, AF and NW were major collaborators and named co-investigators.
